# Commentary: Gut and oral microbiome profiles in patients with obesity and ischemic heart disease

**DOI:** 10.3389/fcimb.2026.1834415

**Published:** 2026-04-27

**Authors:** Yiman Huang, Yongyan Tang, Yiling Huang, Yan Wang

**Affiliations:** 1 The Eighth Affiliated Hospital of Sun Yat-sen University, Shenzhen, Guangdong, China; 2 Health Care Center Of Shenzhen International Travel, Shenzhen, Guangdong, China

**Keywords:** 16S rRNA sequencing, gut microbiome, IHD, obesity, oral microbiome

## Introduction

Understanding the intricate dialogue between the human microbiome and host metabolism is a frontier in biomedical research, particularly for complex diseases like obesity and ischemic heart disease (IHD). The study by Starodubova et al., (2025) ventures into this compelling territory, presenting a comparative analysis of gut and oral microbiota in patients stratified by obesity and IHD status. By concurrently examining these two distinct microbial niches, the authors provide a broader, albeit more complex, snapshot of the microbial landscape associated with cardiometabolic disorders. Their work rightly underscores the premise that microbial dysbiosis may be a contributory factor and a potential therapeutic target. However, as we delve deeper into the nuances of microbiome research, several critical considerations emerge from their findings, which warrant thoughtful discussion to guide future research towards more definitive mechanistic insights and clinically actionable conclusions.

## Considerations on study design and confounding variables

While the study adeptly identifies distinct microbial signatures associated with obesity and IHD, particularly in the co-morbid Obesity-IHD group, the interpretation of these findings must be contextualized within the study’s design. A primary consideration is the inherent challenge of confounding variables in observational microbiome studies. The authors appropriately note limitations such as unequal group sizes, age disparities, and differential statin use—with 76% of the Obesity-IHD group on statins compared to 30% in the Control group. Statins themselves may modulate the gut microbiome, presenting a confounder that is difficult to fully dissect (Dharmarathne et al., 2024). This situation highlights a fundamental dilemma in observational studies. The key factors that act as confounders—such as diet and medication—are also among the most powerful modulators of the microbiome itself. Future research must therefore not only statistically adjust for these confounders but also proactively characterize their effects. Study designs that incorporate detailed longitudinal tracking of medication initiation/dosage and employ validated dietary questionnaires or metabolomic profiling are crucial. This will help distinguish whether a microbial signature is a core component of the obesity-IHD pathophysiology or a secondary reflection of its management. Furthermore, although exclusion criteria removed individuals on antibiotics or probiotics three months prior, long-term dietary patterns, which are profound drivers of microbial composition, were not controlled for, as no detailed dietary or lifestyle data were reported in the study methodology. The observed microbial shifts, such as reductions in *Parabacteroides* and *Flavonifractorin* the Obesity-IHD group, could be influenced by these unmeasured dietary or pharmacological factors. Consequently, these observed microbial shifts may not be intrinsic to the disease states per se, and the identified taxa are better interpreted as context-dependent biomarkers associated with obesity-IHD, rather than as definitive etiological agents of its pathogenesis.

## The challenge of establishing causality and the oral-gut axis

The cross-sectional design of the study, while effectively revealing associations, inherently limits causal inference. For instance, the intriguing correlations between specific bacteria (e.g., *Blautia* spp. with higher BMI) raise the pivotal but unanswered question: are these microbial alterations drivers of disease or consequences of it? To untangle this temporal relationship and establish directionality, future longitudinal or interventional studies are essential. Moreover, the study’s parallel analysis of oral and gut microbiomes presents a unique opportunity to speculate on the often-hypothesized “oral-gut axis.” The authors found decreased oral *Alloprevotella* and *Capnocytophaga*in the Obesity-IHD group. It should be noted that the current study did not report detecting these or other classic oral taxa (e.g., *Porphyromonas*) within the gut microbial profiles, which would be a more direct evidence of translocation.​Alternatively, do periodontal pathogens that transiently translocate to the gut induce trained immunity in gut-resident immune cells, creating a persistent pro-inflammatory state? Interventional studies in animal models, where oral microbiota is selectively manipulated, coupled with simultaneous profiling of systemic inflammation markers and gut immune responses, are required to validate such mechanistic pathways. Recent reviews highlight that oral pathogens like *Porphyromonas gingivalis*can translocate and perturb gut barrier function, creating a pro-inflammatory loop (Niu et al., 2024). While the current data cannot confirm such a pathway, framing the observed oral changes within this systemic axis model could strengthen the biological plausibility of the findings and suggest novel mechanisms linking oral health to cardiovascular outcomes.

## Integrating findings into a mechanistic framework and future directions

**Figure 1:**
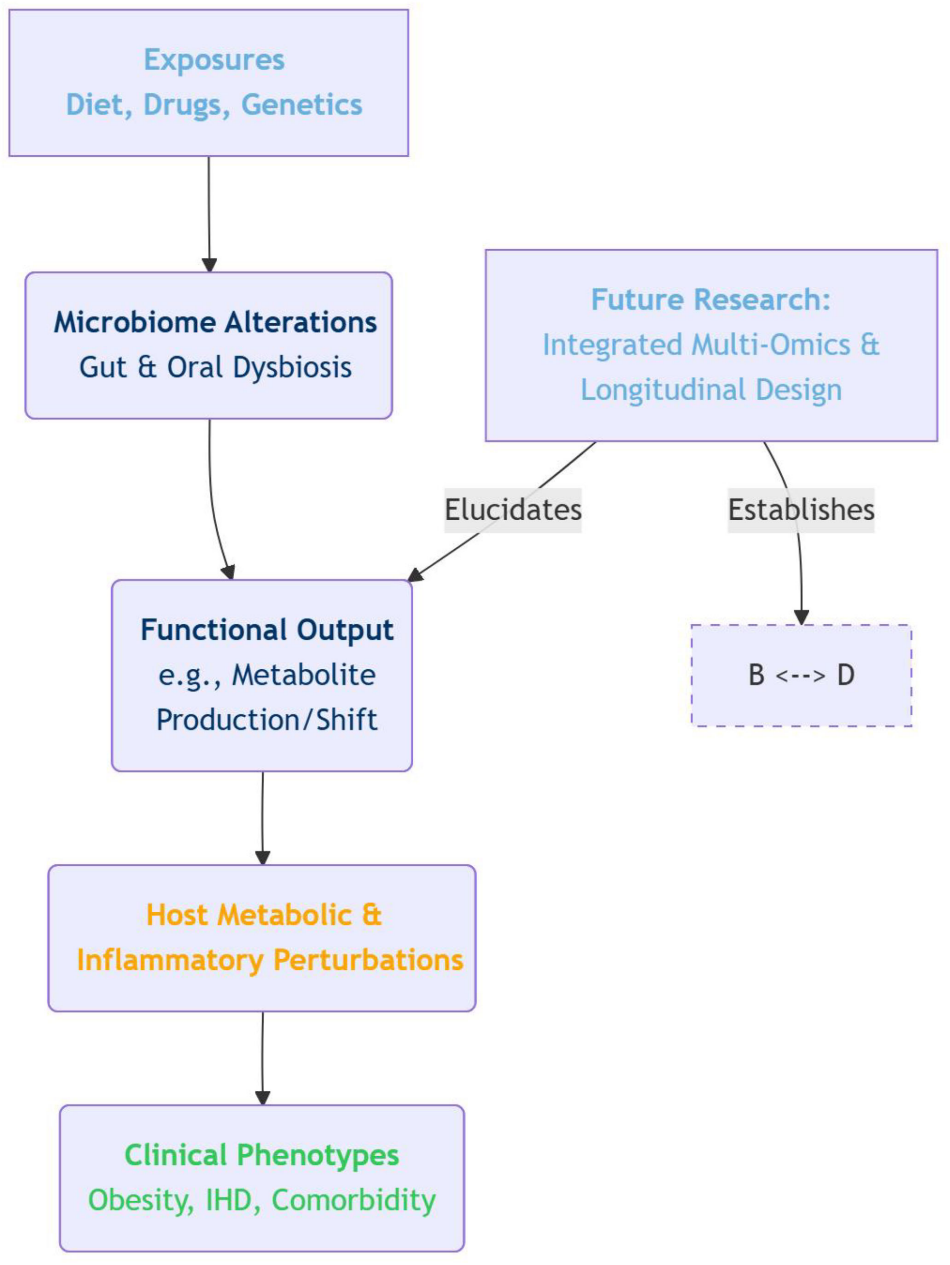
Conceptual framework linking microbiome alterations to cardiometabolic disease and future research priorities.

The true translational potential of microbiome research lies in moving beyond cataloguing associations to elucidating function. A direct strategy is to move from 16S rRNA gene surveys to metatranscriptomics and metabolomics of the same samples. This allows researchers to ask not just “which bacteria are there?” but “what are they doing?” and “what molecules are they producing in the disease state?”. Identifying conserved microbial metabolites (e.g., specific bile acid derivatives or imidazole propionate) that correlate with disease across diverse cohorts offers more tractable therapeutic targets than bacterial taxa themselves. This is because metabolites, unlike specific bacterial species, can be directly inhibited or supplemented through pharmacological or nutritional interventions. The study identifies several genera of interest (e.g., *Streptococcus*, *Intestinibacter*, *Agathobaculum*) that are elevated in the Obesity-IHD gut microbiome. The critical next step is to understand *what these microbes are doing*. Do these taxa produce metabolites like trimethylamine N-oxide (TMAO) or specific short-chain fatty acids (SCFAs) that directly impact host inflammation, endothelial function, or lipid metabolism? Integrating metagenomic sequencing to predict functional potential or coupling microbiome profiling with metabolomic analysis of serum or feces would dramatically enhance the biological insight gained from 16S rRNA data. Such an integrated multi-omics approach could transform a list of differentially abundant bacteria into a coherent narrative about disturbed microbial metabolism contributing to disease. In this commentary, we propose a conceptual framework to visually synthesize the core hypothesis and the suggested future research path emerging from this analysis. The framework is illustrated below (**Figure 1**).

This schematic illustrates the proposed pathway from environmental and host exposures to the clinical phenotypes of obesity and ischemic heart disease (IHD), mediated through microbiome alterations and their functional outputs. The model emphasizes the central role of mechanistic investigation (e.g., microbial metabolite profiling) and highlights the critical need for integrated multi-omics and longitudinal study designs to establish causal links and advance the field beyond observational associations.

Furthermore, the pursuit of microbial biomarkers for diagnostics or therapy must contend with immense inter-individual variability. The lack of significant differences in alpha diversity between groups in this study echoes the inconsistent findings in the literature, suggesting that beta diversity (community structure difference) and specific taxonomic changes may be more reliable signals than overall richness. Future studies should aim to define conserved, pathogenic microbial ‘functions’ (e.g., the induction of trained immunity) to develop robust universal targets (Hajishengallis and Chavakis, 2021). This approach is likely to be more fruitful than relying solely on specific bacterial taxa, which show high inter-individual variability. While the integration of metatranscriptomics and metabolomics represents the ideal path forward, the substantial cost and sample size requirements for such comprehensive multi-omics studies must be acknowledged. A pragmatic, stepwise approach may be necessary, especially in initial phases or resource-limited settings. A cost-effective first step could involve leveraging functional prediction tools (e.g., PICRUSt2, Tax4Fun2) on existing 16S rRNA data to hypothesize which metabolic pathways are enriched or depleted. Subsequently, targeted assays for key microbial metabolites implicated in cardiometabolic disease—such as short-chain fatty acids (SCFAs), specific bile acid derivatives, or trimethylamine N-oxide (TMAO)—could be performed on available biospecimens. This targeted metabolomics approach can validate functional predictions and establish mechanistic links with greater feasibility than untargeted discovery metabolomics. Such a strategy allows for the prioritization of resources towards the most promising functional hypotheses before committing to larger-scale, untargeted multi-omics analyses.

## Discussion

In conclusion, Starodubova et al. provide a valuable comparative profile that reinforces the association between distinct gut and oral microbial communities and the comorbid state of obesity and IHD. Their work successfully identifies several candidate bacterial genera warranting further investigation. Building upon their findings, the conceptual framework proposed herein highlights the necessity of integrating multi-omics and longitudinal approaches to translate these associations into mechanistic understanding. The primary academic contribution of this study is the parallel documentation of ecological shifts in two microbial habitats, strengthening the evidence for systemic dysbiosis in cardiometabolic disease. The key guidance for future research emanating from this commentary is a call for greater integration. Isolating the specific effects of confounders like medication, employing longitudinal designs to infer causality, and, most importantly, integrating genomic and metabolomic data to uncover mechanism are essential next steps. By embracing these more complex, functional analyses, we can better assess whether modulating the specific microbial communities identified in the original study (e.g., *Streptococcus, Intestinibacter, Agathobaculum* which were elevated, or *Parabacteroides, Flavonifractor, Alloprevotella, Capnocytophaga* which were reduced), perhaps through personalized probiotics, prebiotics, or dietary interventions, holds genuine promise for breaking the link between obesity and its devastating cardiovascular complications.
